# Supplementary figures and images for: Translocation of benzo(a)pyrene reactive metabolites across human mammary epithelial cell membranes
Source: PLoS One. 2025 Dec 3;20(12):e0337395. doi: 10.1371/journal.pone.0337395 (PMC12674524; doi:10.1371/journal.pone.0337395)

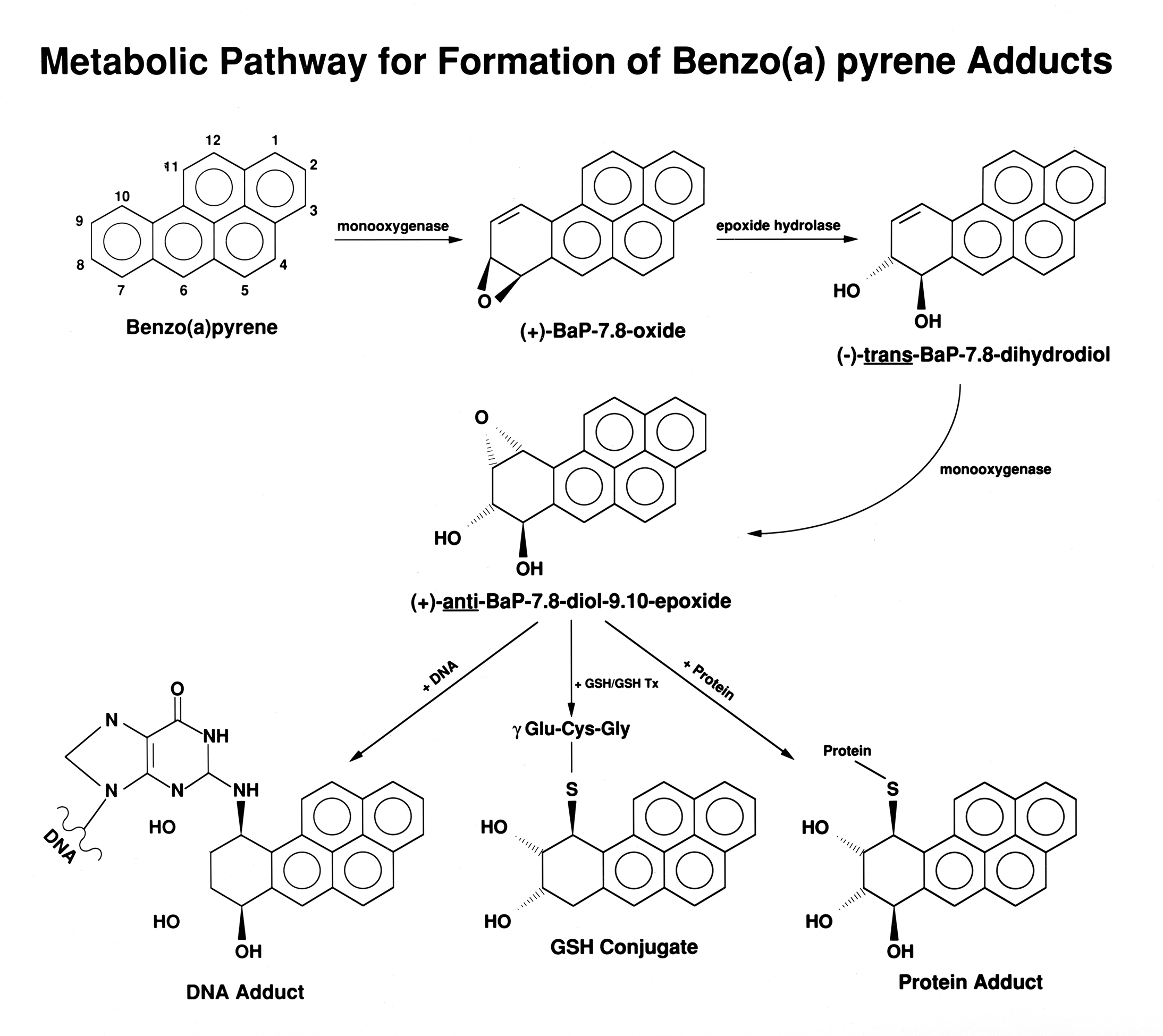

Supplement: S1 Fig — Metabolic pathway for BaP adduct formation of macromolecules is shown. Other BaP electrophiles may be involved in adduct formation as well. Enzymatic GSH conjugation of BaP is a detoxification pathway. (TIF) [file pone.0337395.s001.tif]

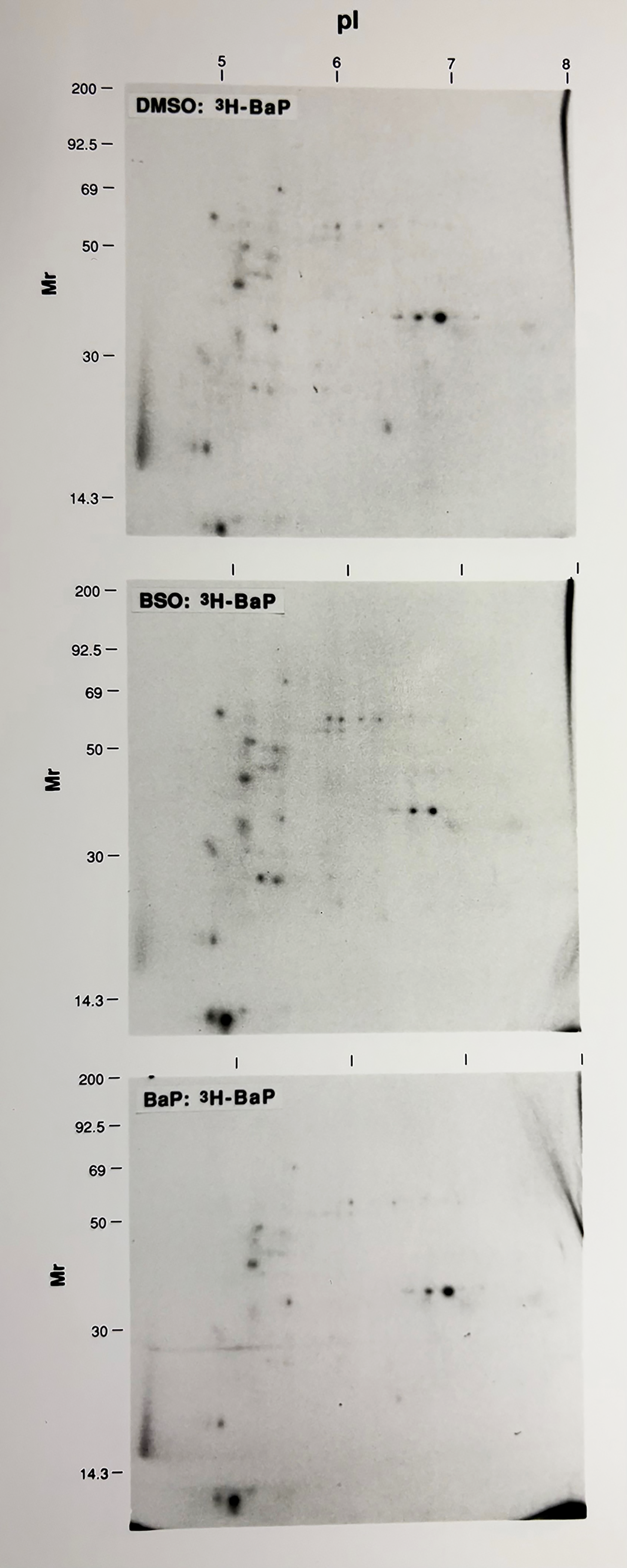

Supplement: S2 Fig — T47D cells were pretreated for 48 hr with 0.5 mM BSO or 4 µM BaP (unlabeled) to decrease or increase GSH compared to DMSO control (0.4 v/v). Pretreated cells were then exposed to 4 µM 3H-BaP (6 Ci/mmole) for 24hr after which cellular protein was collected, acetone precipitated, washed to remove unbound radioactivity, lyophilized and then solubilized according to methods. A 500,000 dpm amount of labeled protein was separated by 2D PAGE, after which gels were fixed, dried and exposed to film for 8 weeks at −80°C. (TIF) [file pone.0337395.s002.tif]
