# Supplementary material for: Translocation of benzo(a)pyrene reactive metabolites across human mammary epithelial cell membranes
Source: PLoS One. 2025 Dec 3;20(12):e0337395. doi: 10.1371/journal.pone.0337395 (PMC12674524; doi:10.1371/journal.pone.0337395)
Supplement: S1 Raw Images — (PDF) [file pone.0337395.s005.pdf]

S1 File. Raw gel and fluorograph images.

Raw Images for Fig 4: BaP extracellular adducts in culture media after separation by 2D-PAGE. The raw 2D-PAGE gel was photographed after silver staining (labeled Silver Stain). The second image (labeled DMSO: 3H-BaP) is a fluorograph from a dried 2D-PAGE gel exposed to x-ray film.

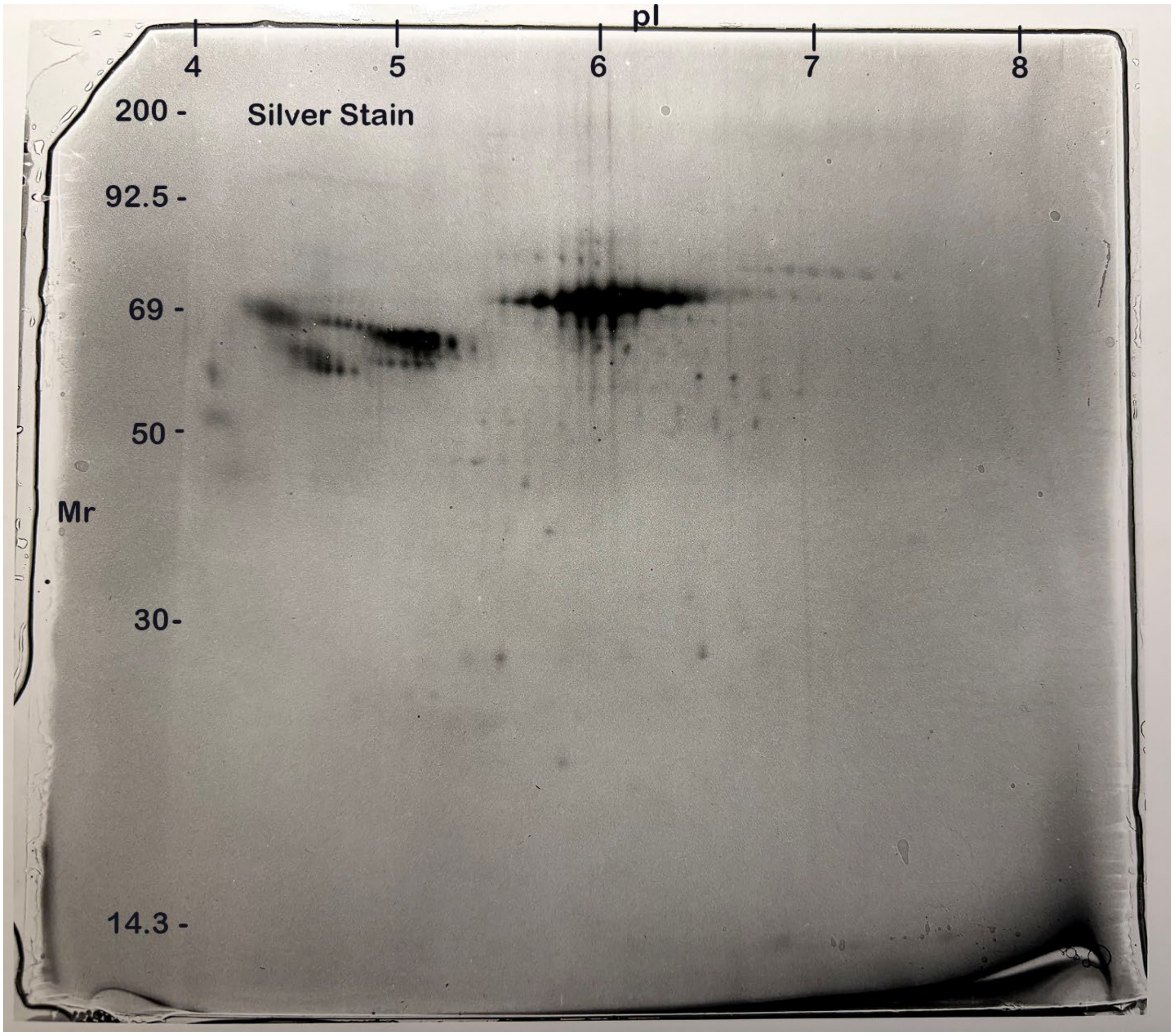

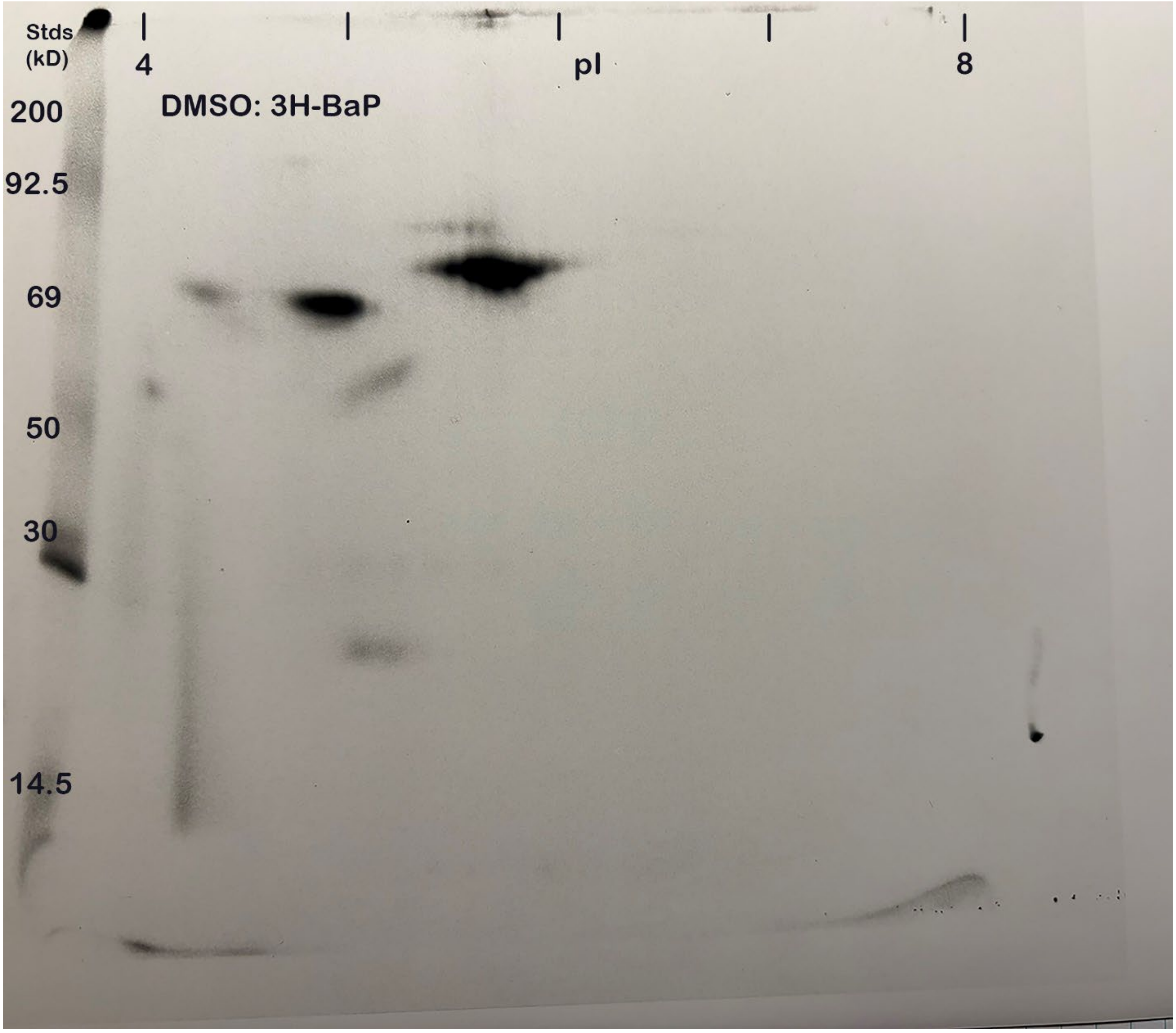
Stds
(kD)
200
92.5
69
50
30
14.5
4
pI
8
DMSO: 3H-BaP

Raw Images for Fig 5: Silver staining and 3H-BaP cellular protein adducts in T47D cells. The raw 2D-PAGE gel was photographed after silver staining (labeled Silver Stain). The second image (labeled 3H-BaP) is a fluorograph from a dried 2D-PAGE gel exposed to x-ray film. White arrow indicates beta-actin.

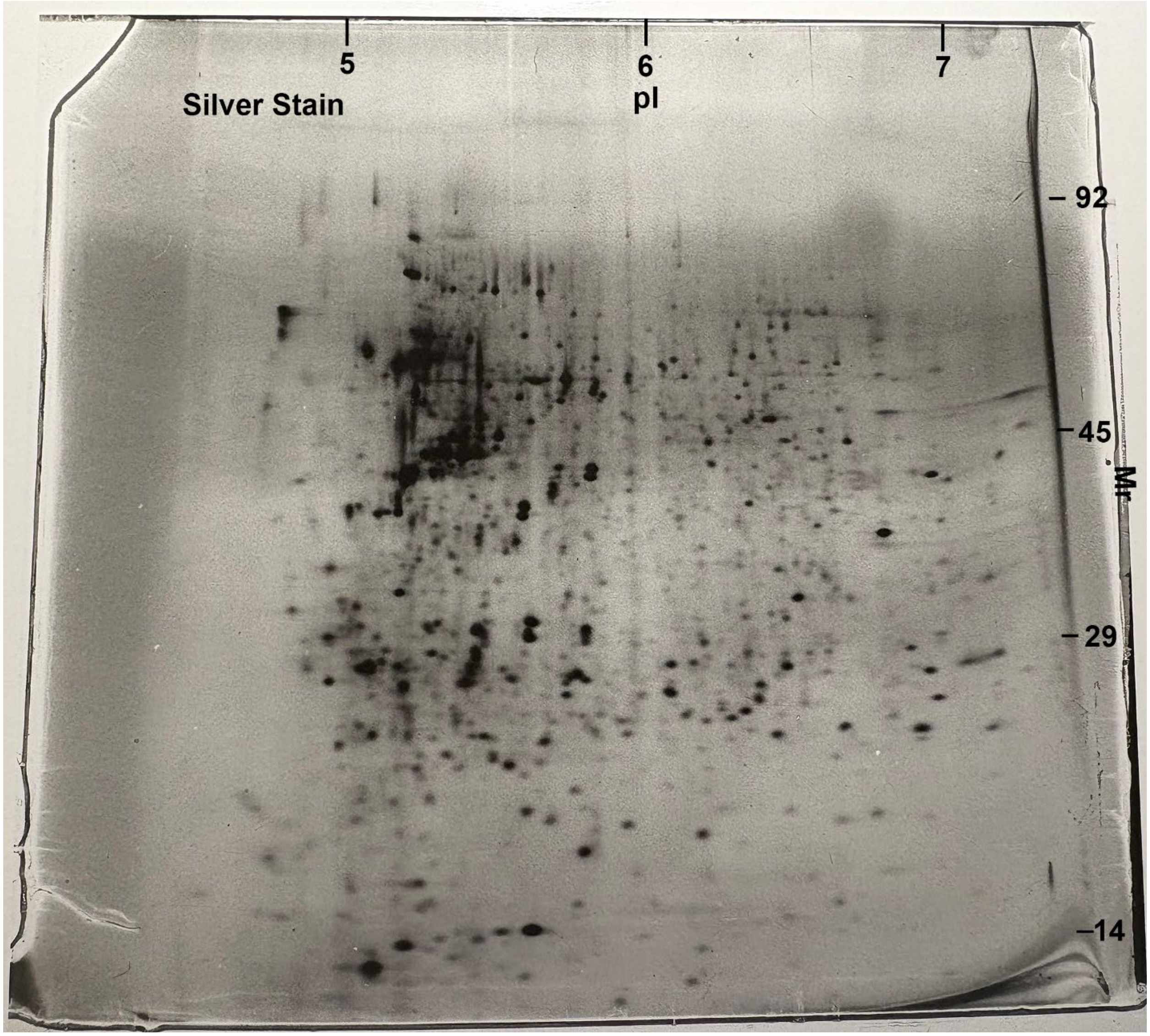

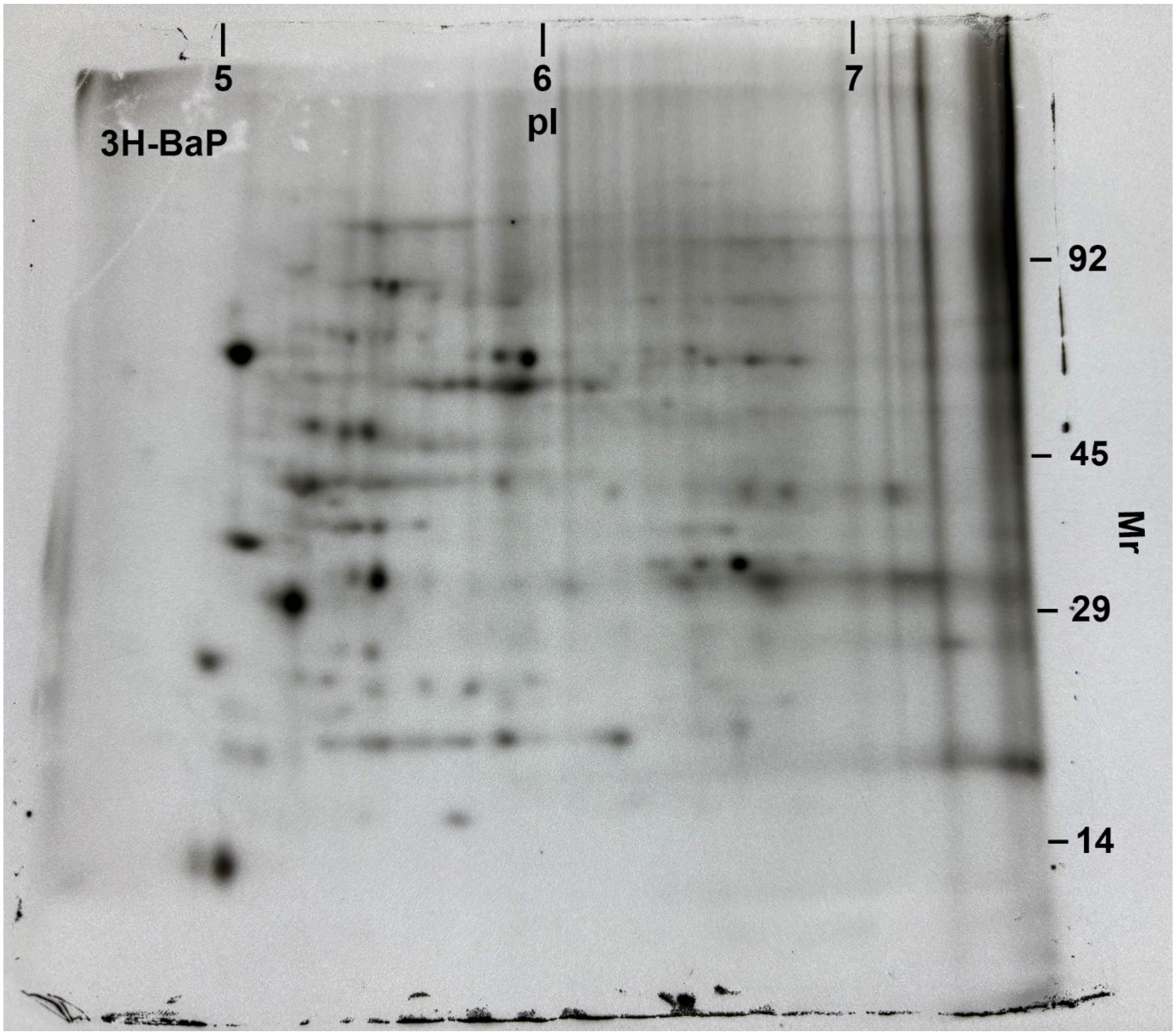

Raw Images for Fig S2_Fig: 3H-BaP cellular protein adducts after DMSO, BSO or BaP. These three images are fluorographs from dried 2D-PAGE gels exposed to x-ray film. Images are labeled, DMSO: 3H-BaP; BSO: 3H-BaP; and BaP: 3H-BaP.

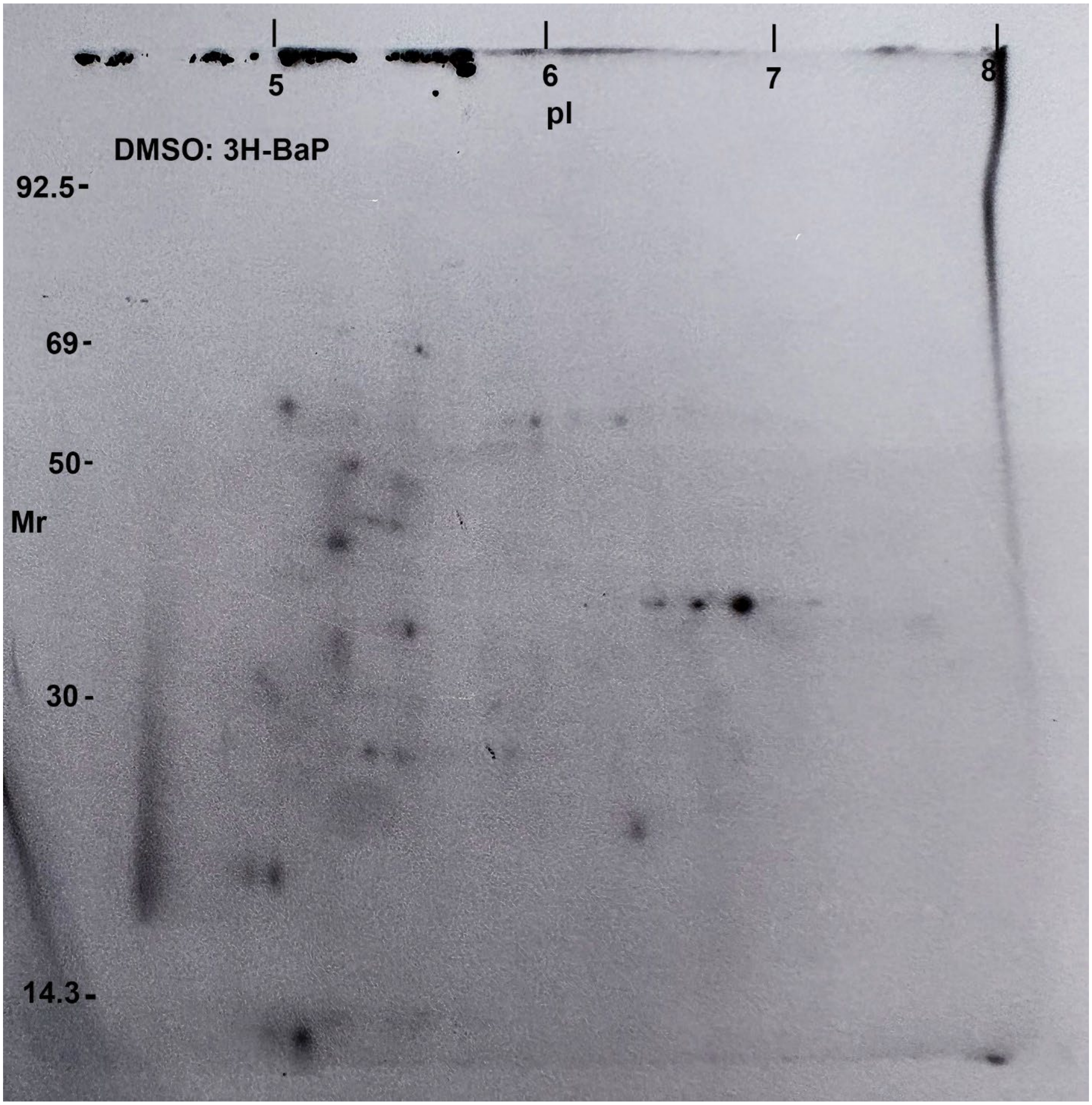

DMSO: 3H-BaP
pI
5
6
7
8
Mr
92.5
69
50
30
14.3

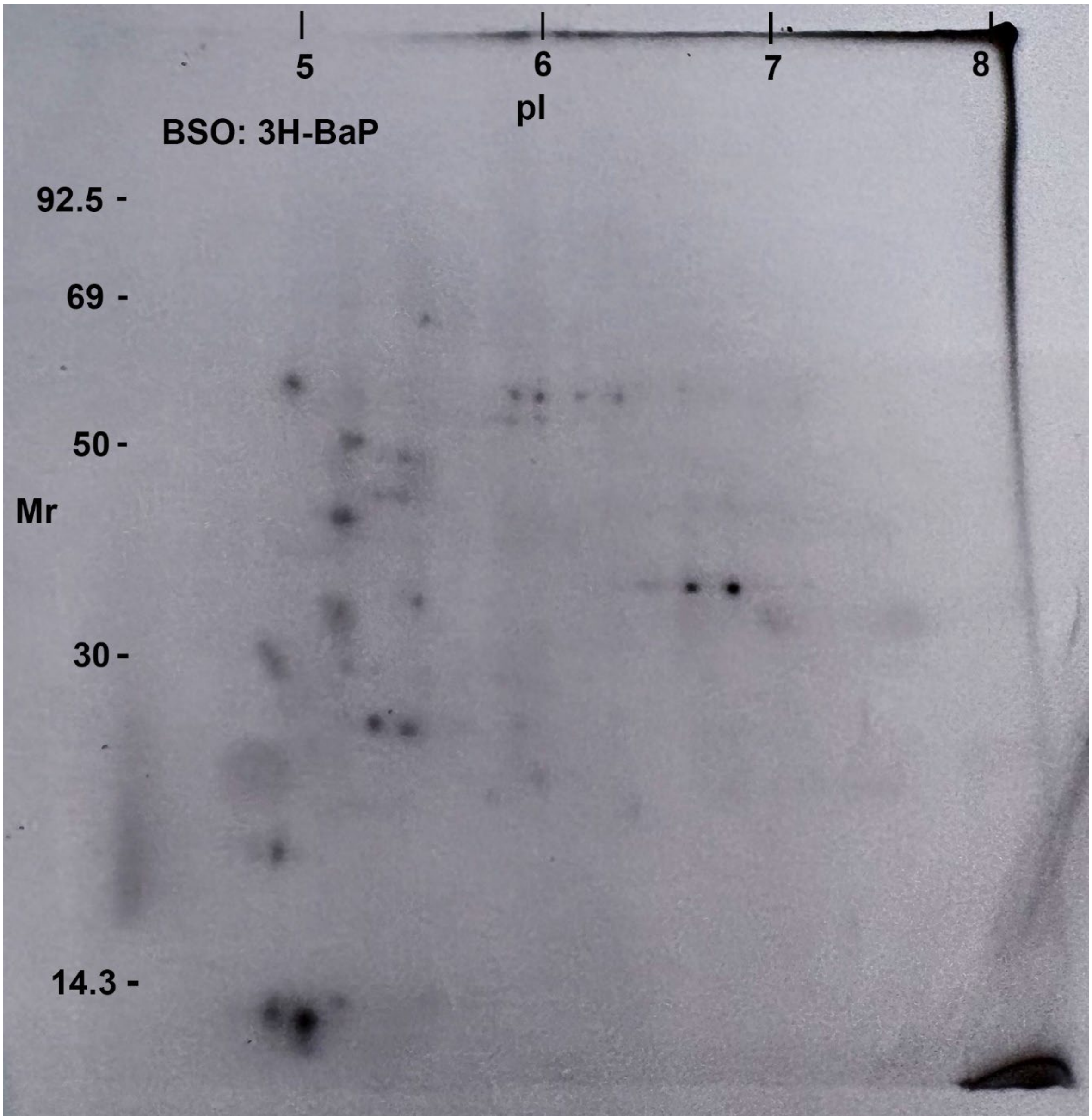
5
6
7
8
pI
BSO: 3H-BaP
92.5 -
69 -
50 -
Mr
30 -
14.3 -

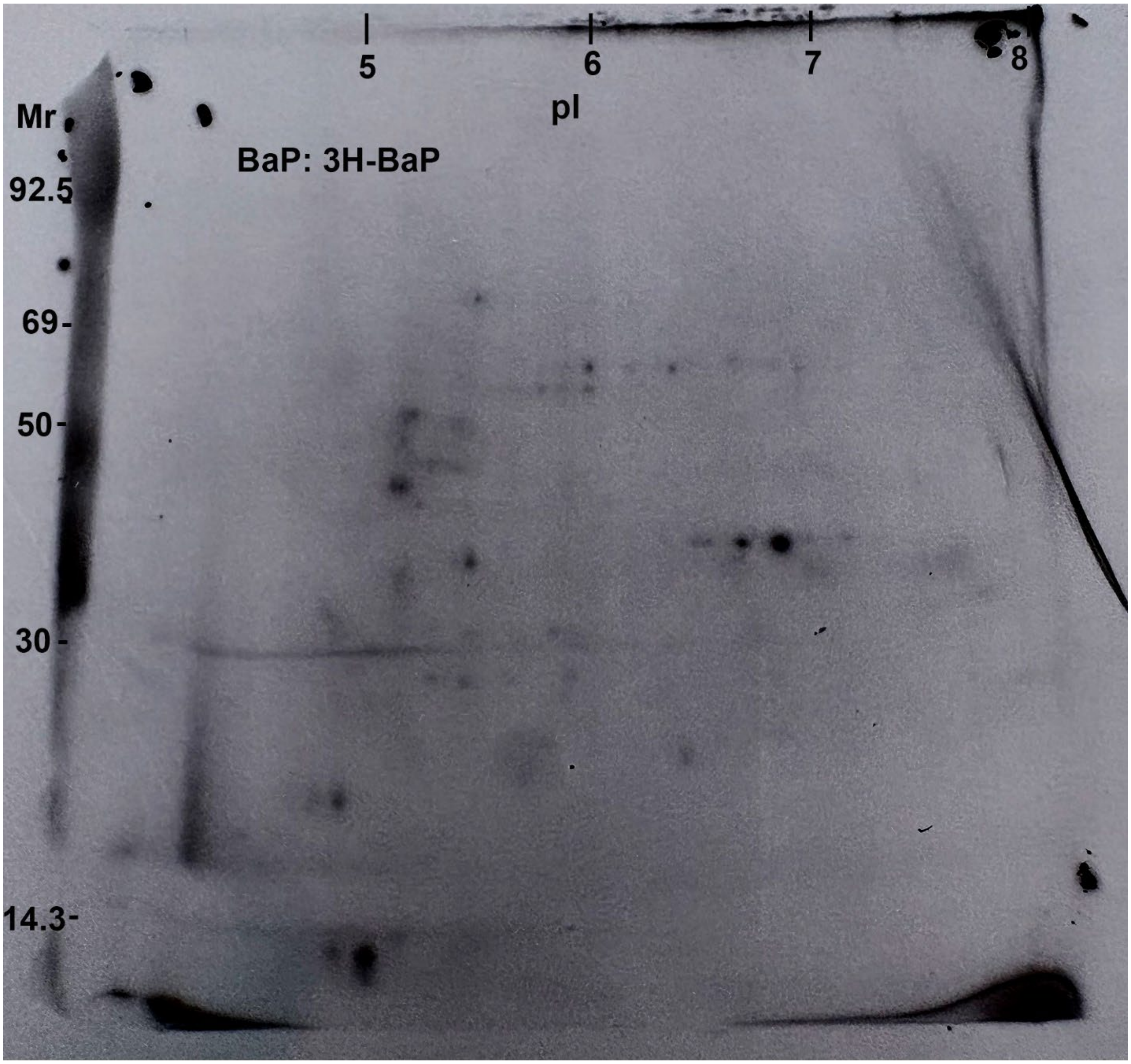

5
6
7
8
pI
Mr
BaP: 3H-BaP
92.5
69-
50-
30 -
14.3-
